# In their own words: a qualitative study of factors promoting resilience and recovery among postpartum women with opioid use disorders

**DOI:** 10.1186/s12884-020-02872-5

**Published:** 2020-03-18

**Authors:** Daisy J. Goodman, Elizabeth C. Saunders, Kristina B. Wolff

**Affiliations:** 1 Dartmouth Geisel School of Medicine, 46 Centerra Parkway, Office 338, Lebanon, NH 03766 USA; 2 The Dartmouth Institute for Health Policy and Clinical Practice, 74 College Street, Vail Building 709, Dartmouth College, Hanover, NH 03755 USA; 3 The Dartmouth Institute for Health Policy and Clinical Practice, Dartmouth College, 46 Centerra Parkway, Lebanon, NH 03766 USA

**Keywords:** Resilience, Pregnancy, Opioid use, Women, Postpartum

## Abstract

**Background:**

Opioid use disorder (OUD) is associated with substantial morbidity and mortality for women, especially during the perinatal period. Opioid overdose has become a significant cause of maternal death in the United States, with rates highest in the immediate postpartum year. While pregnancy is a time of high motivation for healthcare engagement, unique challenges exist for pregnant women with OUD seeking both substance use treatment and maternity care, including managing change after birth. How women successfully navigate these barriers, engage in treatment, and abstain from substance use during pregnancy and postpartum is poorly understood. The aim of this study is to explore the experiences of postpartum women with OUD who successfully engaged in both substance use treatment and maternity care during pregnancy, to understand factors contributing to their ability to access care and social support.

**Methods:**

We conducted semi-structured, in-depth interviews with postpartum women in sustained recovery (*n* = 10) engaged in a substance use treatment program in northern New England. Interviews were analyzed using grounded theory methodology.

**Results:**

Despite multiple barriers, women identified pregnancy as a change point from which they were able to develop self-efficacy and exercise agency in seeking care. A shift in internal motivation enabled women to disclose need for OUD treatment to maternity care providers, a profoundly significant moment. Concurrently, women developed a new capacity for self-care, demonstrated through managing relationships with providers and family members, and overcoming logistical challenges which had previously seemed overwhelming. This transformation was also expressed in making decisions based on pregnancy risk, engaging with and caring for others, and providing peer support. Women developed resilience through the interaction of inner motivation and their ability to positively utilize or transform external factors.

**Conclusions:**

Complex interactions occurred between individual-level changes in treatment motivation due to pregnancy, emerging self-efficacy in accessing resources, and engagement with clinicians and peers. This transformative process was identified by women as a key factor in entering recovery during pregnancy and sustaining it postpartum. Clinicians and policymakers should target the provision of services which promote resilience in pregnant women with OUD.

## Background

Prenatal substance use is a persistent public health problem in the United States. An estimated 8.5% of pregnant women nationally report non-medical drug use, with a sharp rise in use of opioids [1]. Between 2002 and 2013, rates of heroin use increased at twice the rate among women as men [2], contributing to a 127% rise in opioid use during pregnancy [3]. The estimated prevalence of perinatal opioid use disorder (OUD) grew from 1.5 to 6.5 per 1000 delivery hospitalizations from 1999 to 2014 [4]. A tragic increase in maternal deaths from opioid-related causes accompanied this increase [3, 5, 6].

The current surge in prenatal OUD has led to a renewed focus on improving care for affected women and infants, including development of national treatment guidelinesand allocation of federal funds aimed at increasing access to appropriate care [7–11]. Despite these efforts, more than one-third of women with documentation of OUD in the year before giving birth do not receive pharmacotherapy, the gold standard treatment for perinatal OUD. Discontinuation of treatment in the first 6 months postpartum is even more common [12].

Pregnant women with substance use disorders are often perceived as criminals rather than individuals with a serious health condition [13], resulting in an uncertain and potentially hostile environment of care [14, 15]. For many pregnant women, substance use treatment is difficult to obtain due to lack of programs willing to treat pregnant women, cost, lack of medical coverage, fear of legal consequences, and threat of child protection involvement for women who have children [16, 17]. These factors intensify the stigma associated with prenatal substance use [16, 18–21] and are reinforced by social determinants, further limiting access to care [19, 22, 23].

Previous research exploring the experience of pregnant women with substance use disorders has focused on structural barriers and vulnerabilities [18, 19, 22–29], butless is known about factors which *facilitate* treatment success. Few published studies have described factors contributing to positive outcomes in this population [30, 31], despite recognition of the importance of employing a strengths-based approach in research about people who use substances [32]. *Resilience* is a term broadly defined as the interactive and dynamic process of adapting, managing, and negotiating adversity. While resilience is used widely to describe individuals’ response to chronic disease, with the concept has particular relevance for substance use and recovery, and their antecedent conditions [32–35]. Individuals must experience stress or adversity before developing resilience, and consequently demonstrate positive adjustment and adaptation to it through ongoing interaction with environmental factors [33]. Resilience is both process-based and evolutionary, rather than a static trait which an individual does or does not have [36].

Gill Windle, a psychologist who studies quality of life, communication, and well-being of people with dementia, conducted a systematic review and concept analysis to clarify the definition of resilience and examine resilience through the perspective of multiple disciplines. Windle identifies four levels of protective factors which play a role in the development of resilience in people with chronic conditions: individual, family/household relationships, neighborhood/social context, and social policy. Exploring both positive and negative interactions between internal and external factors across levels provides insight into how a person adapts to and manages adversity [32].

Increasing rates of perinatal substance use and opioid-related maternal mortality [3] underscore the urgency of engaging pregnant and postpartum women in effective treatment [6, 37–39]. There is a critical need to identify factors which promote initiation as well as continuation of treatment after birth. Therefore, our study explored the experiences of postpartum women with OUD who engaged in both substance use treatment and maternity care during pregnancy, to learn about barriers *and* facilitators which contributed to the ability to achieve recovery in the face of personal and structural challenges.

## Methods

### Procedures

Between 2015 and 2016 we conducted in-depth, semi-structured interviews with postpartum women (*n* = 10) enrolled in a program providing substance use treatment for pregnant and postpartum women in northern New England. Participants were recruited during treatment visits through verbal invitations and a written brochure. Interested participants were offered a choice between being interviewed by someone they knew through the program (DG), or a nurse educator unknown to them. All chose to be interviewed by DG, except one who had no preference. Interviews were approximately 60 min in length, including open questions about participants’ substance use history, experience with prenatal care and OUD treatment, and barriers and facilitators to initiating and remaining in care (Additional file 1). Interviews were conducted until theoretical saturation was reached, defined as the point at which no new constructs were emerging, with regard to our research questions [40].

Due to the vulnerability of participants, the consent process was anonymous, particpants provided verbal and written consent. A non-identifiable study number was assigned to each participant, used to label the recorded interview, and noted on the consent form. All interviews were digitally recorded, stored on an encrypted flash drive, transcribed verbatim by a member of the study team (ES), and verified by DG. Recordings were destroyed after transcription.

### Analysis

The HyperRESEARCH program [41] was used to facilitate data analysis. Two *a priori* codes, “barriers” and “facilitators”, were established before the coding process began. Using grounded theory methods, open coding and iterative constant comparison were used from the onset of the coding process to identify conceptual similarities and differences within the data to establish codes and discover themes [42–44]. Two researchers (ES, KW) independently coded 20% of the interviews and met for consensus sessions until greater than 85% concurrence was reached. The remaining interviews were coded independently. Codes and subcodes were derived inductively and revised iteratively. Themes and subthemes were developed by each researcher independently and disagreements settled via consensus (Table 1).

**Table 1:** Examples of coding for each theme

Quote	Code Examples	Subcode Examples*	Themes
“And the first thing I thought of was, “Oh, I’m gonna eff this baby up. Like, I need to like stop what I’m doing”. It literally was like one of the first things I thought of.” (Participant 10)	Patient pregnancy	- Feelings about pregnancy - Relationship of participant& baby - Impact of SUD/OUD on others	Pregnancy as a Change Point
“You know, told her what was going on. It was really hard, but I knew that um … You know, it was all or nothing and I, I’ve gotta trust somebody. I’ve gotta tell someone so that my baby’s okay. So it kinda took a lot, but um, I felt comfortable with her and you know.” (Participant 8)	Interactions: patient and provider	- Sources of motivation - Feelings about addiction - Stigma/judgement	Seeking Help
Uh, it really like … I felt, I actually while being in this treatment and I have their psychologist here and stuff. And while being in the treatment I actually turned it around. And I was like, “Look, I’m not ready to see my parents now that I’m clean.” (Participant 7) Mhm. (Interviewer) ‘Cause I don’t want them to jeopardize this! So it actually turned around for me. And, and once they did start tryin’ to come around, I was like, “I’m not ready. I’m not ready, I don’t want you to jeopardize, you know, this.” (Participant 7)	Interactions: family & friend	- Sources of motivation - Treatment experiences - Trust / Lack of trust	Develop Self-Efficacy
“So, I wish things were open on the weekends! I don’t work on the weekends! (Participant 6) (Laughs) That would be better, I agree! I think we do need to deal with the fact that people have jobs and it’s more difficult to get in.” (Interviewer) “Right, that’s a problem.” (Participant 6):	Barriers: system	- Coordination of care - Financial - Support	Agency and Selfcare
“So I think the more, more knowledge to younger people and then maybe more advertising, like I was saying, more advertising like at WIC …. Like in the bathrooms, or something like that..” (Participant 4) “Oh, good suggestion. Absolutely, that’s a good suggestion.” (interviewer) "Just so if people do need help. ‘Cause most women aren’t going to be to their OB/GYN, “Hey, you know, I use pills every day. Can you help me?” … It’s hard, it’s like you’re behind yourself, watching yourself say it to the doctor. It’s just … once you say it, then it’s like a big … weight lifted off your shoulders. (Participant 4)	Facilitators	- Feelings about treatment /addiction - Support - Communication	Caring for Others

## Results

### Participants

Ten women who had entered treatment for OUD during pregnancy and remained engaged in treatment during the postpartum period were interviewed between 2 weeks and 1 year after delivery. Participants had a mean age of 28 years, identified their race/ethnicity as non-Hispanic White or multiracial (White/Native American), and had a diagnosis of OUD. Five had initiated prenatal care during the first trimester, the remainder during the second or third trimesters. Half were first time mothers. All had been referred to the treatment program by prenatal providers and continued to receive care in the same obstetric clinic. All participants were receiving buprenorphine for the treatment of OUD.

#### Engagement in care before pregnancy

Before becoming pregnant, participants experienced barriers which affected access to substance use treatment. The majority lacked insurance coverage before becoming pregnant, and primarily used the emergency department for medical care. Participants reported that they were unable to enter treatment due to long waiting lists at OUD treatment programs, lack of insurance, and transportation problems. These barriers felt insurmountable, leaving women with little choice but to continue using illegally obtained opioids. One participant reported unsuccessfully contacting multiple treatment providers: “I’d tried calling … maybe a year, 9 months to a year before [becoming pregnant], only to be told: ‘We have a waiting list,’ or ‘Your insurance doesn’t cover,’ and it just seemed like there were no avenues” [Participant 4].

Several participants attended intake appointments at opioid treatment programs which required cash payments, but were unable to afford the cost of attendance or medication. Therefore “at the time, it was more reasonable to keep using because it was cheaper” (Participant 8). Some had not sought treatment prior to pregnancy. However, with diagnosis of pregnancy, women became eligible for Medicaid and were immediately able to cover the cost of treatment attendance and medication for OUD.

### Pregnancy as change point

At the individual level, discovering that they were pregnant caused a fundamental shift in women’s motivation to seek treatment. The diagnosis of pregnancy was perceived as the inflection point. According to one participant: “It just felt like a perfect opportunity to stop … to better myself and to do better and just be the person that I know I could be … it felt kinda like a wakeup call. Like, here’s your opportunity” (Participant 4). Another said: “Just finding out that I was pregnant did give me hope. It made me feel like, wow, I really have – not just for myself- but I have a reason to stop” (Participant 7). Many described an abrupt change in focus from their own needs to concern about risk to the fetus. For example, one woman described her resolve to avoid withdrawal symptoms which could cause fetal harm: “Yes, she changed everything. And I had two kids, but … it’s different when they’re out and you know what you’re doing. I mean, it is hurting them, but not physically hurting them. But then to have a baby inside of you, everything you do hurts them” (Participant 5).

### Seeking help

Women also described a shift in their sense of agency and self-efficacy, resulting in efforts to self-manage substance use while they sought treatment. Women did their best to reduce harm based on available knowledge, utilizing social networks and web-based resources to obtain information. After her pregnancy was diagnosed during an emergency room visit, Participant 7 described how: “Me and my boyfriend had done our own research after leaving the hospital. Immediately we were on the phone, Googling what to do with an addiction problem and being pregnant.”

Another explained:

I found out I was pregnant. I continued using Percocets [oxycodone] for about a month. And then, from my prenatal care and my people on the street and friends, I heard about Suboxone [buprenorphine/naloxone]. So, I took myself off of the Percocets, switched myself to the Suboxone [buprenorphine/naloxone]... and made it work until I could get in … So, I guess I tried to play my own doctor and tried to do what was right. (Participant 1)

Her experience was not unique. Based on information from associates or the internet, other participants also reported obtaining the treatment medication buprenorphine illegally, believing this was safest during pregnancy.

All participants confronted the need to disclose substance use to maternity care providers in order to access treatment. While most chose to do this verbally, one intentionally provided a urine sample containing non-prescribed buprenorphine as a way to inform her midwife. “I told [the midwife] right away. I had some in my system, and I actually did it on purpose … She goes, ‘I found Subutex [buprenorphine] in your system.’ I said, ‘I know. I take that for a reason’” (Participant 2).

Disclosure was an intense and emotional moment for most participants, who feared stigma, legal consequences, and child protective services involvement. Nevertheless, motivated by the desire to prevent harm, women gathered courage: “It’s like, oh my God, I have to go in there and tell [the clinician] I was a drug addict,” (Participant 6). Some described a sense of relief afterwards: “I just felt a … rush and just was like, I have to say it. And I kind of felt, like an out of body experience saying it. Then afterwards I was like, ‘Oh my’. And then I was like, ‘I can’t take it back!’” (Participant 4). Only Participant 9 denied that this conversation with providers was challenging: “I’m a really open person. And I find that when I’m more open with the doctors, they do what needs to be done.”

Responses from maternity care providers were varied, ranging from perceived rejection through support. Although some women initially sought maternity care from midwives or at community hospitals where they had delivered other children, they were immediately referred to a high-risk obstetric service because of their substance use. This caused a loss of pre-existing relationships with trusted local providers.

The nurse was like, ‘Are you using drugs?’ and I was like ‘Yeah, I’m using opiates’ … And then they were like, ‘Oh, well, we don’t really have the resources to deal’, it kind of made me feel like I was, like ‘Oh my God, we can’t deal with you’ type. So it made me feel like a disease. Like a contagious disease. (Participant 4).

Other providers were unexpectedly supportive and helpful, providing reassurance and immediate referrals to treatment.

And [the maternity provider] was like, ‘Okay, let’s get you some help’. And I’m like, ‘There’s people out there that would actually want to do that?’ You know? And she’s like, ‘Um, yeah! You know, you’re pregnant and you guys get like first priority. And so you’ll get in there in two weeks.’ And I was just like, ‘Two weeks?’ I was expecting a number more like two months. (Participant 8).

This uncertainty surrounding provider response served both as a barrier to seeking care, and an unexpected moment of growth for participants who asked for help and received it.

### Developing self-efficacy

Throughout pregnancy and treatment engagement, participants described an evolution of self-efficacy with regards to managing OUD. Participants acknowledged an overall shift in their relationship to substance use. Upon learning she was pregnant, Participant 7 told her partner: “Things need to change because this is not the way we can live. We’re living very, very harmfully … We gotta start reachin’ out. We gotta start being honest. We can’t keep hiding this. So, from then on we decided this was it.” While many women initially felt shame (i.e. Participant 4, above), some also developed self-acceptance and a new sense of competence with regards to recovery:

But now I feel like I beat my addiction, I am bettering myself. So I feel … You know, I just feel like a lifetime ago. I feel like, *was that really me?* But I’m so proud of myself, you know, that one day in the hospital with the OB to say, ‘Yes. Yes I do. And please get me help.’ (Participant 4)

Another noted: “You know, people fall and make mistakes. But you can bounce back. … .. it’s not the end of the world to make a mistake, but how you react afterwards and pick yourself up is the important part” (Participant 3). This self-efficacy reinforced women’s efforts to engage with and remain in treatment during and after pregnancy.

### Agency and self-care

Increasing self-efficacy also contributed to women’s ability to engage in self-care, from managing difficult relationships, through overcoming logistical barriers and accessing needed resources. As confidence grew, some made consequential decisions about social relationships with partners, family members, and friends. This often involved losing connections with former associates when they entered treatment. “Because we didn’t have drugs anymore, nobody wanted to help. You know, all those favors we did, they didn’t matter anymore” (Participant 7). Others described how a shift in motivation and renewed commitment to self-care led to decisions about ending non-supportive relationships, including with partners who continued using drugs. Although this sometimes resulted in loss of transportation or housing, participants identified these decisions as necessary for success. Participant 7 described her need to avoid family members who disapproved of her engagement in a program which used medication to treat OUD during pregnancy. “I’m not ready to see my parents now that I’m clean. Cause I don’t want them to jeopardize this!” Another recounted ending her relationship with the father of her children, who pressured her to share her treatment medication and caused her prescription to run out early.

It would be hard for me because I’d be short at the end … and then it’d be in my head, like ‘What’s more important? Trying to make sure I have enough for me and the baby, or for this grown-ass man that’s not getting help and crying to me like a baby?’ We actually ended up splitting up because it got to the point that I said, ‘I’m done … I’m not supporting your habit. And you need to get help. If I can get help, why can’t you?’ So I left him. (Participant 4)

Concern for her pregnancy became a catalyst to allow her to care for herself.

While some participants ended non-supportive relationships, others chose to accept support from friends and family. Participant 8 described receiving unexpected assistance and encouragement from her mother-in-law. “I was lucky that my husband’s mother has been very supportive throughout the whole thing. She would help me do research and stuff. And she was one of those people you would think wouldn’t be supportive, based on who she is as a person and amongst society. But she turned out to actually be my biggest advocate.” For some, family and friends provided both emotional support and assistance for navigating barriers to recovery. Participant 10 explained that her boyfriend’s support was instrumental. “[My boyfriend] just always made me feel very comfortable with whatever I was doing, like ‘We’ll figure it out.’ Like when I stopped working … I was like eight months pregnancy, crying ‘cause they [coworkers] were just ridiculous, and he’s like, ‘We’re done … If I work a bunch of overtime, we’ll figure it out.’”

Participants also developed effectiveness in overcoming logistical challenges. Attending appointments for both prenatal care and substance use treatment involved managing transportation, time, and financial constraints. This required organizational skills. “It was just like appointment after appointment. My weeks were packed full … I worked two jobs … So I was a busy girl. I got a big planner. Jotting everything down, oh my God, it’s packed full still with her appointments and mine!” (Participant 2). Another took steps to rearrange her work schedule so she could attend weekly treatment appointments. “I have to go in at 4:30 am because I’m calling out [to go to treatment]” (Participant 6). Navigating treatment successfully required developing new skills and persistence. “I had to change myself first. I had to say, ‘No, don’t set on [delay] this. You need to get it done, you need to do this’” (Participant 2).

### Caring for others

Improved capacity for self-care contributed to the desire to care for others. Attending a substance use treatment group with other women was described by several participants as a significant source of support and information, enhancing confidence about childbirth and the care of a newborn likely to experience opioid withdrawal. Participant 2 explained: “I was worried about the birth. Group really helped with that because there were a lot of girls who had just recently had their babies, and they shared their experience with us and it really kind of eased your mind that your baby was gonna be okay.” Others described how group members provided mentorship to each other: “We all bounce things off of each other, like, we’re giving each other ideas and we’re giving each other praise” (Participant 10). Women linked their own recovery to their support for one another: “It just seemed like a buddy system … And it’s nice to feel like you’re paying it forward. Like, this is where I was, I’ve been there, don’t feel bad about yourself, because you’re doing the best you can” (Participant 4). Peers also provided a sense of accountability: “If you do use [drugs], and then you gotta come tell the group and you feel bad and you don’t want to do that, so then that stops you” (Participant 9). Though most were unequivocally positive about the support of peers, two participants worried about being judged by other group members. Participant 10 perceived some group members as being “very judgmental of each other,” although Participant 8 felt that her anxiety about being judged decreased with time. “There’s nothing like an intimate setting and having to be honest and speak publicly … I think I still start feeling warm and get red in the face when it’s my turn to talk, but now that I’m one of those people that have been here awhile … I don’t feel quite so looked at or judged...” (Participant 8).

Participants also reached out to women in their home communities, bringing them to the treatment program, and helping them establish care. Women described feeling fortunate to be in treatment and recovery: “I’m thankful that I’m here, and at this point in time, I’m happy where I am, I don’t want to change a thing. But I do, in a sense, wish that I could leave so somebody else could come in, and have a spot and have the help that they need. But me being selfish, I don’t want to leave” (Participant 1). Another explained that she agreed to be interviewed in order to help others: “That’s why this research is so important. Because … there’s just so many things that need to be changed and done.” (Participant 7).

## Conclusions

Despite formidable barriers, women participating in this study achieved positive outcomes through engaging in prenatal care and treatment during their recent pregnancies. Their success in maintaining recovery postpartum provides valuable insight into factors contributing to the development of resilience among women with OUD. Key themes emerging from interviews included the significance of pregnancy as a change point, the difficult but liberating choice to disclose treatment need to obstetric providers, the emergence of agency over substance use and relationships associated with it, the development of self-care strategies utilizing available community and social policy-level supports, and the desire to give back to others. An overarching theme of resilience emerged, which developed through the interaction between women’s inner motivation and her ability to utilize or positively transform external factors.

Themes developed through our analysis significantly align with the individual, family/household, social/community, and social policy levels described in Windle’s framework. Windle describes how the interplay of personal assets (strengths), protective factors and challenges contribute to the development of resilience among people with long term health conditions [33] (Table 2). This model can be used as a heuristic device through which treatment programs and policymakers can identify ways to optimize support for perinatal women with OUD.

**Table 2:** Emergent themes by Windle’s levels of protective factors

Emergent Themes	Illustrative Quote
**Individual level**	
Pregnancy as catalyst for shift in motivation for treatment	“I needed something like an anchor, like the pregnancy because, I don’t know, I’d probably still be using to this day.” (Participant 6)
Self-management and harm reduction strategies while awaiting care	“I had known someone that was in this program and I knew that they prescribed the buprenorphine. And so that’s what I was finding on the street, because I knew that’s what they would give me.” (Participant 10)
Making the choice to disclose treatment need	“When I made my appointment I said, ‘I’m pregnant, I’m an addict, can you take me?’ It was actually a big relief.” (Participant 1) “I was an emotional mess, but like, it was the nurse! ‘Cause you know, the list of regular questions that they have to ask. And one of the questions is, “Do you use drugs?” and I started bawling. I was like, ‘Yes, that’s why I’m here,’ and she’s like, ‘It’s all right, it’s all right, you can talk more about it when the doctor comes in.’ Like, she was super nice about it as well. I had planned on telling them on my own. But then when she asked, I was like, oh well this makes it much easier. I can just tell them the truth. Like, that’s what I’m here to do … So that I could get the help that I needed.” (Participant 10)
Development of self-efficacy and agency	“The other day, I really wanted to smoke a bowl with a couple of friends of mine. But I was like, no. For once it actually felt empowering to do something different. To do what no one else is doing. Like, the unpopular thing. It felt good to be a different person … I’m not gonna screw that up again because I’m doing so well.” (Participant 8)
Move toward self-acceptance	“I felt like the biggest piece of shit in the world for a while. I was depressed about it [using while pregnant] for a long time. And then I was like, ‘Well, it is what it is. No changing it.’” (Participant 6)
Development of self-care strategies utilizing available supports	“The transportation issue is a big thing … The Medicaid ride brought me a lot of the time … The driver gave me his cell phone number to call, just in case, ‘cause I told him I’d had problems before [getting left at appointments].” (Participant 3)
**Family/household level**	
Making decisions about relationships	“At the beginning that’s what you have to do. You have to stay away from everybody and all your friends and just not be around it. Especially if you’re not pregnant and want to get clean, because there’s nothing inside of you saying, ‘Don’t do it, because if you do you’re gonna hurt me.’ If you just want to get clean for yourself, I think you should stay away from it for like a good six, seven months.” (Participant 5) “I was actually clean on my own for a good while … And then, I don’t know what it was … I think it was things were going on in our relationship, our marriage … It was almost like my subconscious telling me, like, ‘Oh my God. To even hang out with this motherfucker you gotta be high.’” (Participant 8)
**Social/community level**	
Peer support	“When you see that these other moms are doing it … it helps to make you that much stronger, to not want to use.” (Participant 9)
Caring for others	“One of my friends comes here now … She was 4 or 5 months pregnant. She was usin’ drugs. And she just didn’t want to deal with it. But it kept gettin’ further and further … And I had given her the number plenty of times, she never called. And … she asked me if I’d give her the number again because she’s showin’ and she really needs to do something. And she was buying Suboxone [buprenorphine/naloxone] off the street. And I said, ‘You should really just go here’. And she said she’s nervous to come into a group by herself. And I told her, ‘I’ll go with you’. So she comes now every week. She’s pregnant and she comes here now.” (Participant 3)
**Social policy level**	
Improved access to resources (e.g., insurance, transportation)	“I looked into it [treatment], but it was all nothing that I could afford. So I just kept doing what I was doing and getting by and I got pregnant and I got my insurance and that’s really helped out.” (Participant 1)

At the individual level, participants described pregnancy as an inflection point at which they experienced intense motivation for substance use treatment. Although women described guilt, anxiety, and a sense of risk when initially seeking help, they also felt relief after disclosing [18]. A strong sense of responsibility contributed to developing a capacity for self-care, including accepting support when available, and also avoiding sabotage at the family/household level. The shift in intrinsic motivation for change which occurred due to pregnancyfueled capacity to access social and material supports. This and confirms previous research that pregnant women are more likely to engage in detoxification, residential, or methadone treatment programs than nonpregnant women [45]. Our findings support the critical role clinicians can play by creating safety for women to disclose their need for treatment and facilitating access to resources. Maternity care providers were recognized by study participants as critical access points for substance use treatment, and programs should therefore consider how to align perinatal care and substance use treatment.

As described by Windle, interactions between internal motivators and factors at the community level also contributed to women’s resilience. Upon entering treatment, women made essential decisions about participating in social networks and relationships. Support from peers was empowering, increasing participants’ confidence in navigating their own care and that of their infants, and promoting a sense of reciprocity and accountability. In this rural New England context, peer support may be a particularly powerful facilitator due to the high risk for social isolation in low resourced rural communities [46]. Providing opportunity for group interaction is therefore an important component of treatment for this population, as well as programmatic elements such as case management and transportation assistance which enhance women’s utilization of available community resources.

Previous research has described the influence of external social pressures such as risk of incarceration or loss of child custody to increase treatment engagement and retention for pregnant women [47]. In contrast, women in the current study entered treatment voluntarily, inspired by a combination of internal motivation and external opportunity (i.e. Medicaid eligibility) provided by a system which had previously denied them access. Study participants described being resigned to continued drug use due to a lack of treatment access prior to pregnancy, resignation which was transformed to a sense of possibility after becoming pregnant and able to access services. As Windle points out, it is the *interaction* between internal motivation and external access to resources which is critical to the development of resilience [48–53].

Limitations to this study include its small, relatively homogeneous, self-selected sample. We intentionally chose to interview women who were currently in treatment and had sustained recovery throughout pregnancy and into the postpartum period. Participants were fairly consistent in their perceptions and offered few dissenting perspectives. Therefore, the experiences of our sample may differ from those of women from other cultural backgrounds, geographic regions, or earlier stages of recovery. Further research should also include women who do not engage in treatment during pregnancy, or who are unable to maintain recovery postpartum.

The majority of participants in this study chose to be interviewed by a staff member (DG) who was known to them clinically. This may have limited the expression of negative feelings about experiences with treatment and recovery. However, trust is acknowledged as an essential component of qualitative research, and prior studies support the effectiveness of known clinicians as interviewers [54]. To avoid introduction of bias during thematic analysis, coding and interpretation was conducted by two researchers (ES, KW) not involved in providing treatment or prenatal care.

Pregnant and postpartum women who use substances face multiple barriers to engaging in and sustaining recovery. Clinicians working with pregnant women with substance use disorders should focus on programmatic elements which enhance self-efficacy and the development of resilience, including those described by participants in this study. Federal and state policies which deny medical coverage to non-pregnant women with substance use disorders have potentially serious implications for women’s ability to sustain recovery postpartum. Programs and policies which provide support at multiple social levels enhance resilience among pregnant and parenting women with substance use disorders, and increase the likelihood of sustained recovery.

## Supplementary information

**Additional file 1.** ‘In their own words’ Interview Guide. Researcher interview guide of questions and prompts for participanat interview

**Additional file 2.** ‘In their own words’ COREQ Checklist. Completed COREQ Checklist fo the ‘In their own words’ study

## Data Availability

The datasets generated and/or analyzed during the current study are not publically available to protect individual participant privacy.

## Abbreviations

OUD: Opioid Use Disorder (p.2)
DG: Daisy J. Goodman (p.6)
ES: Elizabeth C. Saunders (p.6)
KW: Kristina B. Wolff (p.7)

## References

[1] Substance Abuse and Mental Health Services Administration (SAMHSA) (2018). Results from the 2017 National Survey on drug use and health: detailed tables.

[2] Office on Women’s Health (2016). Opioid use, misuse, and overdose in women.

[3] Maeda A, Bateman BT, Clancy CR, Creanga AA, Leffert LR (2014). Opioid abuse and dependence during pregnancy: temporal trends and obstetrical outcomes. Anesthesiology..

[4] Haight SC, Ko JY, Tong VT, Bohm MK, Callaghan WM (2018). Opioid use disorder documented at delivery hospitalization - United States, 1999-2014. Morb Mortal Wkly Rep.

[5] Metz TD, Rovner P, Hoffman MC, Allshouse AA, Beckwith KM, Binswanger IA (2016). Maternal deaths from suicide and overdose in Colorado, 2004-2012. Obstet Gynecol.

[6] Schiff DM, Nielsen T, Terplan M, Hood M, Bernson D, Diop H (2018). Fatal and nonfatal overdose among pregnant and postpartum women in Massachusetts. Obstet Gynecol.

[7] Substance Abuse and Mental Health Services Administration (SAMHSA). Clinical guidance for treating pregnant and parenting women with opioid use disorder and their infants. Rockville: US Department of Health and Human Services; 2018. Contract No.: HHS Publication No. (SMA) 18–5054.

[8] American College of Obstetricians and Gynecologists (ACOG), American Society of Addiction Medicine (ASAM) (2017). Opioid use and opioid use disorder in pregnancy: Committee opinion No. 711. Obstet Gynecol.

[9] Reddy UM, Davis JM, Ren Z, Greene MF (2017). Opioid use in pregnancy, neonatal abstinence syndrome, and childhood outcomes: executive summary of a joint workshop by the Eunice Kennedy Shriver National Institute of Child Health and Human Development, American College of Obstetricians and Gynecologists, American Academy of Pediatrics, Society for Maternal-Fetal Medicine, Centers for Disease Control and Prevention, and the March of Dimes Foundation. Obstet Gynecol.

[10] World Health Organization (WHO) (2014). Guidelines for identification and management of substance use disorders in pregnancy.

[11] Alliance for Innovation on Maternal Health (AIM) (2017). Obstetric care for women with opioid use disorder.

[12] Wilder C, Lewis D, Winhusen T (2015). Medication assisted treatment discontinuation in pregnant and postpartum women with opioid use disorder. Drug Alcohol Depend.

[13] Terplan M, Kennedy-Hendricks A, Chisolm MS (2015). Prenatal substance use: exploring assumptions of maternal unfitness. Subst Abus.

[14] Oberman M (1992). Sex, drugs, pregnancy, and the law: rethinking the problems of pregnant women who use drugs. Hastings Law J.

[15] Inciardi JA, Lockwood D, Pottiger AE (1993). Women and crack-cocaine.

[16] Roberts SC, Pies C (2011). Complex calculations: how drug use during pregnancy becomes a barrier to prenatal care. Matern Child Health J.

[17] Howard H (2016). Experiences of opioid-dependent women in their prenatal and postpartum care: implications for social workers in health care. Soc Work Health Care.

[18] Stringer KL, Baker EH (2018). Stigma as a barrier to substance abuse treatment among those with unmet need: an analysis of parenthood and marital status. J Fam Issues.

[19] Taylor OD (2010). Barriers to treatment for women with substance use disorders. J Hum Behav Soc Environ.

[20] Krans EE, Patrick SW (2016). Opioid use disorder in pregnancy: health policy and practice in the midst of an epidemic. Obstet Gynecol.

[21] Stone R (2015). Pregnant women and substance use: Fear, stigma, and barriers to care. Health Justice.

[22] Allen K (1995). Barriers to treatment for addicted African-American women. J Natl Med Assoc.

[23] Jackson A, Shannon L (2012). Barriers to receiving substance abuse treatment among rural pregnant women in Kentucky. Matern Child Health J.

[24] Appel PW, Ellison AA, Jansky HK, Oldak R (2004). Barriers to enrollment in drug abuse treatment and suggestions for reducing them: opinions of drug injecting street outreach clients and other system stakeholders. Am J Drug Alcohol Abuse.

[25] Copeland J (1997). A qualitative study of barriers to formal treatment among women who self-managed change in addictive behaviours. J Subst Abus Treat.

[26] Denton WH, Adinoff BH, Lewis D, Walker R, Winhusen T (2014). Family discord is associated with increased substance use for pregnant substance users. Subst Use Misuse.

[27] Greenfield SF, Brooks AJ, Gordon SM, Green CA, Kropp F, McHugh RK (2007). Substance abuse treatment entry, retention, and outcome in women: a review of the literature. Drug Alcohol Depend.

[28] Haller DL, Knisely JS, Elswick RK, Dawson KS, Schnoll SH (1997). Perinatal substance abusers: factors influencing treatment retention. J Subst Abus Treat.

[29] Schempf AH, Strobino DM (2009). Drug use and limited prenatal care: An examination of responsible barriers. Am J Obstet Gynecol.

[30] Shahram SZ, Bottorff JL, Oelke ND, Kurtz DL, Thomas V, Spittal PM (2017). Mapping the social determinants of substance use for pregnant-involved young aboriginal women. Int J Qual Stud Health Well Being.

[31] Stajduhar KI, Funk L, Shaw AL, Bottorff JL, Johnson J (2009). Resilience from the perspective of the illicit injection drug user: an exploratory descriptive study. Int J Drug Policy.

[32] Rudzinski K, McDonough P, Gartner R, Strike C (2017). Is there room for resilience? A scoping review and critique of substance use literature and its utilization of the concept of resilience. Subst Abuse Treat Prev Policy.

[33] Windle G (2011). What is resilience? A review and concept analysis. Rev Clin Gerontol.

[34] Herrman H, Stewart DE, Diaz-Granados N, Berger EL, Jackson B, Yuen T (2011). What is resilience?. Can J Psychiatr.

[35] Luthar SS, Cicchetti D, Becker B (2000). The construct of resilience: a critical evaluation and guidelines for future work. Child Dev.

[36] Earvolino-Ramirez M (2007). Resilience: a concept analysis. Nurs Forum.

[37] Rudd RA, Aleshire N, Zibbell JE, Gladden RM (2016). Increases in drug and opioid overdose deaths: United States, 2000-2014. Morb Mortal Wkly Rep.

[38] Centers for Disease Control and Prevention (CDC) (2017). Drug overdose death data.

[39] O'Donnell JK, Halpin J, Mattson CL, Goldberger BA, Gladden RM (2017). Deaths involving fentanyl, fentanyl analogs, and U-47700 - 10 states, July-December 2016. Morb Mortal Wkly Rep.

[40] Saunders B, Sim J, Kingstone T, Baker S, Waterfield J, Bartlam B (2018). Saturation in qualitative research: exploring its conceptualization and operationalization. Qual Quant.

[41] Researchware I (2009). HyperRESEARCH 2.8.3.

[42] Glaser B, Strauss A (1967). The discovery of grounded theory; strategies for qualitative research.

[43] Charmaz K (2006). Constructing grounded theory: a practical guide through qualitative analysis.

[44] Boeije H (2002). A purposeful approach to the constant comparative method in the analysis of qualitative interviews. Qual Quant.

[45] Daley M, Argeriou M, McCarty D (1998). Substance abuse treatment for pregnant women: a window of opportunity?. Addict Behav.

[46] Gjesfjeld CD, Weaver AC, Schommer K (2012). Rural women's transitions to motherhood: understanding social support in a rural community. J Fam Soc Work.

[47] Ondersma SJ, Winhusen T, Lewis DF (2010). External pressure, motivation, and treatment outcome among pregnant substance-using women. Drug Alcohol Depend.

[48] Saleebey D, Saleebey D (2006). The strengths approach to practice. The strengths perspective in social work practice.

[49] Karoll BR (2010). Applying social work approaches, harm reduction and practice wisdom to better serve those with alcohol and drug use disorders. J Soc Work.

[50] Olmstead T, White WD, Sindelar J (2004). The impact of managed care on substance abuse treatment services. Health Serv Res.

[51] Kroll-Desrosiers AR, Crawford SL, Moore Simas TA, Rosen AK, Mattocks KM (2016). Improving pregnancy outcomes through maternity care coordination: a systematic review. Womens Health Issues.

[52] Larson A, Berger LM, Mallinson DC, Grodsky E, Ehrenthal DB (2019). Variable uptake of Medicaid-covered prenatal care coordination: the relevance of treatment level and service context. J Community Health.

[53] Anderson AC, Chen J (2019). ACO affiliated hospitals increase implementation of care coordination strategies. Med Care.

[54] Jack S (2008). Guidelines to support nurse-researchers reflect on role conflict in qualitative interviewing. Open Nurs J.

