# Supplementary material for: In their own words: a qualitative study of factors promoting resilience and recovery among postpartum women with opioid use disorders
Source: BMC Pregnancy Childbirth. 2020 Mar 18;20:178. doi: 10.1186/s12884-020-02872-5 (PMC7081623; doi:10.1186/s12884-020-02872-5)
Supplement: Supplementary file 1 — Additional file 1. ‘In their own words’ Interview Guide. Researcher interview guide of questions and prompts for participanat interview [file 12884_2020_2872_MOESM1_ESM.docx]

**‘In their own words’: Interview Guide**

**Participant ID # ______**

Wks. pregnant when gave birth _______

Wks pregnant when realized she was pregnant _______

Age of this baby now _______

First baby - yes no Cigarettes – yes no Drug used the most _________

**Tell me about realizing you were pregnant and how that came about**

**Tell me about going to the OB clinic while you were pregnant.**

How many visits did you have before giving birth?

What was the biggest barrier to getting care?

What things made (or could have made) getting care easier?

**Let’s talk about using drugs while you were pregnant, can you share some of that experience with me?**

Did you tell your obstetrical provider?

Tell me about that –

When in the pregnancy did you tell them? How did that go?

**Were you worried that using would affect your pregnancy?**

How about smoking?

**Were you in treatment during this pregnancy?**

Did you have problems getting into a program? What was the biggest barrier?

Is there anything you can think of that would have made it easier?

**How about your family? Are you able to talk to them about this?**

**What about your partner? What does he/she think?**

**Tell me about your hospital stay after you gave birth**

**Did you come back to the OB clinic after your baby was born?**

If so, did you choose to start any birth control? If so, are you still using that method?

**Before you knew you were pregnant, when was the last time you were able to get health care?**

**If you could say anything you wanted to your OB providers, what would it be?**
