# Supplementary material for: In their own words: a qualitative study of factors promoting resilience and recovery among postpartum women with opioid use disorders
Source: BMC Pregnancy Childbirth. 2020 Mar 18;20:178. doi: 10.1186/s12884-020-02872-5 (PMC7081623; doi:10.1186/s12884-020-02872-5)
Supplement: Supplementary file 2 — Additional file 2. ‘In their own words’ COREQ Checklist. Completed COREQ Checklist fo the ‘In their own words’ study [file 12884_2020_2872_MOESM2_ESM.docx]

**COREQ: 32 Item Checklist**

| No | Item | Guide questions/description | Page number |
| --- | --- | --- | --- |
| **Domain 1: Research team and reflexivity** | | |  |
| Personal Characteristics | | |  |
| 1. | Interviewer/facilitator | Daisy J. Goodman | 1 |
| 2. | Credentials | DNP, MPH, CNM, CARN-AP | 1 |
| 3. | Occupation | Assistant Professor of Obstetrics and Gynecology, Dartmouth Geisel School of Medicine | 1 |
| 4. | Gender | Female | N/A |
| 5. | Experience and training | Trained in qualitative and quantitative research via MPH and DNP programs and experience in previous research projects | 1 |
| Relationship with Participants | | |  |
| 6. | Relationship established | yes | 6 |
| 7. | Participant knowledge of the interviewer | Participant knew of interviewer via the program; one interviewer | 6 |
| 8. | Interviewer characteristics | Role in treatment program, risk for bias | 6, 20 |
| **Domain 2: Study design** | | |  |
| Theoretical framework | | |  |
| 9. | Methodological orientation and theory | Grounded theory | 6-7 |
| Participant selection | | |  |
| 10. | Sampling | Convenience | 6 |
| 11. | Method of approach | Verbal invitation and a brochure | 6, 20 |
| 12. | Sample size | 10 | 7 |
| 13. | Non-participation | Participants self-selected to be interviewed; volunteered to be part of the project via posted flyers. No one dropped out or withdrew consent for being included in the study. | 6, 20 |
| Setting | | |  |
| 14. | Setting of data collection | Private space associated with medical center: location is not noted in the paper to protect participants’ identities | n/a |
| 15. | Presence of non-participants | No one else was present, except participant’s child in some instances | 6, 20 |
| 16. | Description of the sample | Postpartum women participants enrolled in a program providing substance use treatment for pregnant and postpartum women | 6 |
| Data collection | | |  |
| 17. | Interview guide | Developed by authors, tested with first interview: information about supplementary file note on page 24. | 24 |
| 18. | Repeat interviews | No repeated interviews | n/a |
| 19. | Audio/visual recording | Interviews were audio recorded; deidentified and password protected. All interviews were erased after they were transcribed. | 6 |
| 20. | Field notes | None taken to protect privacy | n/a |
| 21. | Duration | One 60-90 minute interview for each participant | 6 |
| 22. | Data saturation | Interviews were conducted until theoretical saturation was reached. | 6 |
| 23. | Transcripts returned | No transcripts were provided to the participants. Transcripts were reviewed for accuracy by DG & ES, before recordings were erased. | 6 |
| **Domain 3: analysis and findings** | | |  |
| 24. | Number of data coders | 2 | 7 |
| 25. | Description of coding tree | Discussed in methods and codes with examples are in Tables 1 & 2 | 7, 25-26 |
| 26. | Derivations of themes | Initial themes of ‘barriers’, ‘facilitators’, ‘people’ and ‘places’ were used at the beginning of coding. All other themes or codes that were created emerged from the coding process (Table 1) | 7, 25-26 |
| 27. | Software | HyperRESEARCH | 7 |
| 28. | Participant checking | Participants did not provide feedback on the results. | n/a |
| Reporting | | |  |
| 29. | Quotations Presented | Each quote is presented with quote marks and participant identified via participant number. | 8-14 |
| 30. | Data and findings consistent | Yes, checked by DG, ES & KW | n/a |
| 31. | Clarity of major themes | All major themes were clearly presented in the results in Table 1 and discussed in the paper | 7-17, 25-29 |
| 32. | Clarity of minor themes | All sub-themes were presented in Table 2 and discussed in the paper. | 7-15, 25-29 |
